# Synthesis and Characterization of Phosphorylated Cellulose Nanocrystals: Exploring Factors for Enhanced Thermal and Colloidal Stability

**DOI:** 10.3390/polym17192581

**Published:** 2025-09-24

**Authors:** Diego López, María Graciela Aguayo, Mario Núñez-Decap, Pablo Reyes-Contreras, Regis Teixeira Mendonça, Isidora Reyes-González, Benjamín Opazo, Fabiola Valdebenito

**Affiliations:** 1 Departamento de Ingeniería de Procesos y Bioproductos, Facultad de Ingeniería, Universidad del Bío-Bío, Avenida Collao 1202, Concepción 4051381, Chile; diego.lopez2001@alumnos.ubiobio.cl (D.L.); benjamin.opazo2001@alumnos.ubiobio.cl (B.O.); 2 Centro Nacional de Excelencia Para la Industria de la Madera (CENAMAD)—ANID BASAL FB210015, Pontificia Universidad Católica de Chile, Vicuña Mackenna 4860, Santiago 7820436, Chile; mnunez@ubiobio.cl; 3 Departamento de Ingeniería Civil y Ambiental, Facultad de Ingeniería, Universidad del Bío-Bío, Avenida Collao 1202, Concepción 4051381, Chile; 4 Laboratorio de Biopolímeros y Materiales Biobasados, Centro de Biotecnología, Universidad de Concepción, Concepción 4030000, Chile; preyesc@udec.cl (P.R.-C.); rteixeira@udec.cl (R.T.M.); 5 Facultad de Ciencias Forestales, Universidad de Concepción, Concepción 4030000, Chile; ireyes2017@udec.cl; 6 Centro de Energía, Universidad Católica de la Santísima Concepción, Concepción 4090541, Chile; fvaldebenito@ucsc.cl; 7 Departamento de Química Ambiental, Facultad de Ciencias, Universidad Católica de la Santísima Concepción, Concepción 4090541, Chile

**Keywords:** cellulose nanocrystals, surface charge, zeta potential, bleached hardwood kraft pulp

## Abstract

Phosphorylated cellulose nanocrystals (P-CNCs) are a superior alternative to conventional sulfuric acid-derived CNCs because of their enhanced thermal and colloidal stability. However, further research is needed to understand the factors influencing their synthesis and properties for advanced material applications. In this study, P-CNCs were synthesized from bleached hardwood kraft pulp (BEKP) using a controlled hydrolysis method involving pretreatment with H_3_PO_4_ followed by reaction with metaphosphoric acid (HPO_3_) and urea. To optimize the process, a full factorial design was employed to evaluate the effects of reaction time (60–90 min) and HPO_3_ concentration (3–4 M). The P-CNCs were characterized using physicochemical, morphological, and thermal analyses. Surface charge densities ranged from 757 to 1993 mmol/kg, with exceptional colloidal stability, as evidenced by zeta potentials ranging from −30.17 to −67.40 mV. Statistical analysis showed that reaction time had a significant main effect on surface charge (*p*-value = 0.0022) and zeta potential (*p*-value = 0.0448), while a significant interaction between reaction time and HPO_3_ concentration was observed when analyzing the surface charge (*p*-value = 0.0097), suggesting a combined effect of these factors on the surface modification of CNC. Crystallinity indices ranged from 63.6% to 71.3%, and the thermal stability exceeded that of the raw material. These findings contribute to a better understanding of the surface modification and stability of P-CNCs and support efforts to sustainably produce functional CNCs for advanced composite applications.

## 1. Introduction

In recent decades, the development of advanced materials has played a leading role in various technological areas, driven by the need to find functional, sustainable, and high-performance solutions to challenges such as energy efficiency, material protection, fire safety, and environmental sustainability. In this context, nanomaterials derived from renewable resources have attracted considerable interest because of their potential to replace petroleum-based materials, thereby contributing to a circular economy with low environmental impact [1,2,3,4]. One of the most promising nanomaterials in this line is cellulose nanocrystals (CNC), which can be obtained by the controlled acid hydrolysis of the crystalline fraction of cellulose, the most abundant biopolymer in the biosphere. CNCs are characterized by their high crystallinity, high aspect ratio, modulus of elasticity comparable to steel (~150 GPa), low density (~1.6 g/cm^3^), biodegradability, and ability to form stable colloidal suspensions in aqueous media, making them particularly attractive as reinforcements for nanocomposites, functional agents in coatings, and smart additives in advanced formulations [5,6,7]. However, the surface chemistry of CNCs obtained by conventional methods, mainly sulfuric acid hydrolysis, imposes significant limitations on their use in more demanding applications. In these cases, the introduction of sulfate groups (-OSO_3_^−^) on the surface confers a negative charge and colloidally stabilizes the suspensions, but also reduces the thermal stability of the material because sulfate groups catalyze their thermal degradation at temperatures below 250 °C, which restricts their use in systems requiring thermal or flame-retardant resistance [8,9,10,11]. To overcome these limitations, several studies have explored alternative methods of chemical functionalization, among which the phosphorylation of CNCs is an efficient strategy for incorporating phosphate groups (-PO_3_H_2_, -PO_3_^2−^) on the nanofibers’ surface. These groups not only maintain the colloidal stability of aqueous suspensions, but also significantly improve thermal resistance, confer affinity for metal cations, and enable the design of materials with flame-retardant properties, antimicrobial activities, and catalytic or adsorbent functionalities [12,13,14]. Therefore, phosphorylated CNCs (P-CNCs) hold great promise for use in sectors such as construction, tissue engineering, and environmental remediation [15,16,17]. The phosphorylation methods of cellulose reported in the literature, such as reactions with phosphoric acid in aqueous media, phosphate salts in the solid phase, and reactions with organic phosphorylating agents, present limitations in terms of reaction efficiency, control over the degree of functionalization, and severe processing conditions [9,18,19,20]. Several studies have investigated synthesis methods, reaction pathways, process variables, and raw materials for the production of P-CNCs, with the aim of understanding their influence on the properties of the resulting material. This reflects the fact that the synthesis of P-CNCs is still in the exploratory phase, with ongoing efforts focused on identifying the most suitable conditions for optimizing their functionalization. Vanderfleet et al. [21] investigated the thermal degradation of cotton-derived CNCs by modifying their physicochemical properties through acid hydrolysis with mixtures of phosphoric and sulfuric acids. They reported that hydrolysis with phosphoric acid (10.9 M) resulted in a significantly lower surface charge density (<5 mmol/kg) compared to CNCs produced with sulfuric acid (5.4 M), which reached approximately 200 mmol/kg. These findings indicate that, despite its higher concentration, phosphoric acid is less effective than sulfuric acid in introducing surface charge because of its lower esterification efficiency. In this context, Kröger et al. [14] developed an improved methodology for producing P-CNCs with a high surface charge, addressing the major limitations of traditional methods, such as poor reproducibility, low degrees of phosphorylation, unsatisfactory yields, and inefficient reaction sequences. Their approach involved the pre-treatment of cellulose fibers with a urea/phosphate mixture, followed by hydrolysis using gaseous HCl, which enabled the production of P-CNCs with a significantly high surface charge ~2000 mmol/kg, exceeding values reported in previous studies. Furthermore, the process was proven to be simpler, more reproducible, and more sustainable by minimizing water consumption and maximizing yield.

Similarly, other studies have employed statistical experimental designs to evaluate the influence of synthesis parameters on the physicochemical properties of P-CNCs [3,9,22]. Etale et al. [3] proposed a two-step phosphorylation strategy that significantly reduced the required urea-to-acid ratio to approximately 1.3:1, compared to the higher ratios (4:1 or 5:1) commonly reported in previous studies. This decoupling approach enabled the production of CNCs with a broad spectrum of surface charge densities (165–1788 mmol/kg), along with zeta potentials reaching up to −57 mV. The study also revealed a nonlinear relationship between urea concentration and surface charge, indicating that lower urea levels promote the formation of cellulose phosphate nanocrystals, whereas higher concentrations favor the generation of cellulose ammonium phosphate, which is less thermally stable. Although the use of metaphosphoric acid to modify biopolymers such as starch or chitin has been explored [23], its application to obtain P-CNCs is still in its infancy, especially considering the variety of raw materials that can be used to produce these nanomaterials. Based on recent advances in CNC phosphorylation, this study investigated the effects of key reaction parameters on the properties of phosphorylated cellulose nanocrystals (P-CNCs) synthesized from bleached hardwood kraft pulp (BEKP). A controlled hydrolysis method was applied using a mixture of metaphosphoric acid and urea, enabling in situ phosphorylation and minimizing the use of aggressive reagents. This approach aims to improve functionalization efficiency while maintaining the structural integrity of the nanocrystals [3,24]. The resulting P-CNCs were characterized with a focus on surface charge, thermal stability, and colloidal behavior, critical features for advanced material applications. This study aimed to address the existing limitations of CNC thermal performance by systematically exploring the effects of reaction time and acid concentration on the degree of phosphorylation and material functionality. By providing new insights into the process, structure, and properties, this study contributes to the design of sustainable nanocellulose-based materials for high-performance applications.

## 2. Materials and Methods

### 2.1. Materials

Bleached Hardwood Kraft Pulp (BEKP), specifically eucalyptus mixes (*Eucalyptus globulus* and *Eucalyptus nitens*), was kindly provided by the Kraft cellulose industry in the Biobío region of Chile. Ortho-Phosphoric acid (H_3_PO_4_, 85% aqueous solution, CAS 7664-38-2, Merck KGaA, Darmstadt, Germany), metaphosphoric acid (HPO_3_, 33.5–36.5% chips, CAS 37267-86-0, Sigma-Aldrich, St. Louis, MO, USA), urea (CO(NH_2_)_2_, >99%, CAS 57-13-6, Merck KGaA, Darmstadt, Germany), sodium chloride (NaCl, >99%, CAS 7740-23-5, Sigma-Aldrich, St. Louis, MO, USA), sodium hydroxide (NaOH, >99%, CAS 1310-73-2, Merck KGaA, Darmstadt, Germany) and hydrochloric acid (HCl, 34–37% Assay, CAS 7647-01-0, Merck KGaA, Darmstadt, Germany) were purchased. Spectra/Por^®^ MWCO dialysis membrane: 12–14 kD (New Brunswick, NJ, USA) was used for washing pretreated cellulose and phosphorylated cellulose nanocrystals.

### 2.2. Synthesis and Isolation of Phosphorylated Cellulose Nanocrystals (P-CNCs)

Phosphorylated cellulose nanocrystals were synthesized following the methodology reported by Etale et al. [3], with slight modifications. Briefly, five grams of BEKP was added to 100 mL of phosphoric acid 0.7 M (85% *v*/*v*) and preheated to 90 °C. The suspension was stirred at 300 rpm for 90 min to ensure homogeneity. To halt the reaction, 200 mL of distilled water was added. The resulting fibers were washed through five centrifugation cycles at 10,000 rpm for 10 min each, dialyzed with distilled water to a neutral pH, and dehydrated to a thick consistency for further use.

To investigate the influence of reaction time and metaphosphoric acid concentration on the obtained P-CNCs, a full factorial design at two levels with two factors was employed using the Design-Expert software, version 13 (Stat-Ease Inc., Minneapolis, MN, USA). A two-factor interaction (2FI) model was applied to assess the main effects and interactions between the selected variables. The independent variables tested at two levels were metaphosphoric acid concentration (3 and 4 M) and reaction time (60 and 90 min), with one replicate per condition. The response variables analyzed were the surface charge and zeta potential of the resulting P-CNCs.

Following pretreatment, phosphorylated cellulose nanocrystals were synthesized according to the experimental conditions defined in the factorial design. Hydrolysis was performed at 140 ± 5 °C in a recirculating system using a round-bottom three-neck flask immersed in an oil bath. The reactions were performed at 140 ± 5 °C in a recirculating system using a round-bottomed three-necked flask immersed in an oil bath. Urea (4 M) was added until melted, followed by the incorporation of 1.0 g of pretreated cellulose and metaphosphoric acid concentration according to each treatment of the design. The reaction mixture was stirred at 300 rpm for a specific period. Then, the reaction was stopped by adding 40 mL of 1 M NaOH. The suspension was centrifuged three times at 10,000 rpm, and the concentrate from the final wash was resuspended in 15 mL of distilled water and sonicated using a probe sonicator for 15 min at an amplitude of 30%. Finally, the P-CNCs were recovered by centrifugation at 3500 rpm for 10 min, and the suspension was dialyzed (Spectra/Por^®^ Dialysis Membrane MWCO: 12–14 KD, New Brunswick, NJ, USA) against distilled water until a neutral pH was attained.

### 2.3. Characterization of P-CNC

#### 2.3.1. Surface Charge Determination by Conductometric Titration

The surface charge of the cellulose fibers and P-CNCs was determined by conductometric titration, quantifying the carboxyl groups present in the samples, following the methods of Camarero Espinosa et al. [24] and Saito and Isogai [25]. For the cellulose fibers, a 0.6 g sample was dispersed in 110 mL of distilled water, and 10 mL of NaCl solution was added to enhance the conductivity. Hydrochloric acid (HCl) was carefully introduced in a controlled manner until the pH level was adjusted to approximately 3, at which point titration and conductivity assessments were performed. Titration was performed using 0.01 M NaOH with constant stirring. For the P-CNC samples, 100 mg of the biomaterial was dispersed in 100 mL of distilled water, followed by the addition of 30 mL of HCl. The titration volume was determined by linear fitting of the titration data, identifying inflection points in the conductivity and pH curves to define the excess base concentration. The carboxyl group content was calculated using Equation (1) [24]:

(1) $$\frac{mmol\;HPO_{4}^{-}}{kg\;cellulose}=\frac{V_{NaOH}\times C_{NaOH}}{m}\times 10^{3},$$

where *V_NaOH_* is the volume (mL) of NaOH consumed, *C_NaOH_* is the concentration of NaOH (0.01 M), and *m* is the mass of the sample (kg). All measurements were performed in triplicate to ensure reproducibility.

#### 2.3.2. Dynamic Light Scattering (DLS) and Zeta Potential

The particle size of P-CNC was measured using a NANOTRAC FLEX nanoparticle size analyzer (Microtrac Inc., York, PA, USA) based on Dynamic Light Scattering (DLS). This technique provides information on the hydrodynamic diameter and particle size distribution of nanocrystals in aqueous suspensions. The zeta potential of the CNCs was determined using a Stabino^®^ II analyzer (Colloid Metrix GmbH, Meerbusch, Germany). For both particle size and zeta potential measurements, suspensions were prepared by dispersing 50 mg of dry CNCs in 100 mL of water, followed by sonication in an ice bath for 15 min at 30% amplitude using a probe sonicator. All analyses were performed in triplicate.

#### 2.3.3. Transmission Electron Microscopy (TEM)

The morphology of the P-CNC was analyzed using a JEM-1200 EX II transmission electron microscope (JEOL Ltd. Tokyo, Japan) operated at an accelerating voltage of 100 kV. A dilute suspension of P-CNC (0.1 wt%) was sonicated in an ultrasonic bath for 10 min to ensure dispersion and prevent aggregation. A 5 μL aliquot of the suspension was deposited onto carbon-coated copper grids (300 mesh) and allowed to dry at ambient conditions. Bright-field images were obtained at magnifications corresponding to 50 and 100 nm scale bars to resolve individual nanocrystals and evaluate their morphology.

#### 2.3.4. Fourier Transform Infrared Spectroscopy—FTIR

The P-CNCs were characterized by Fourier-transform infrared spectroscopy (FTIR) using an FT-IR spectrometer IRSpirit Series (Shimadzu, Kyoto, Japan) with the potassium bromide pellet technique using freeze-dried NCC samples. For this purpose, 50 mg of freeze-dried NCC was ground in a mortar with 100 mg of potassium bromide, dried for 24 h at 103 °C, and then pressed to obtain pellets. The FTIR spectra were analyzed in the spectral region between 4000 and 400 cm^−1^, with a resolution of 4 cm^−1^ and 32 scans.

#### 2.3.5. X-Ray Diffraction (XRD)

The supramolecular characteristics of the nanocrystals were determined using X-ray diffraction (XRD). Freeze-dried P-CNC pellets (50 mg) were prepared using a hydraulic pellet press. The pellet was arranged in a sample holder, and diffractograms were collected on a D4 Endeavor X-ray diffractometer (Bruker AXS GmbH, Karlsruhe, Germany) with monochromatic Cu Kα radiation (λ = 0.154 nm) at 40 kV and mA. The range over which the intensities were measured was 5° < 2θ < 45°, with scan steps of 0.02°. Curve fitting was performed using the PeakFit, version 4.12 software (Systat Software Inc., San Jose, CA, USA) and Gaussian models to obtain the deconvolution of the peaks. Background correction was performed using OriginPro 9.0 (64-bit) software (OriginLab Co., Northampton, MA, USA) by subtracting the baseline from the diffractogram via interpolation. For each diffractogram, curve fitting was performed, assuming a broad band as the amorphous contribution to the peak broadening. The crystallinity index (Crl) of the samples was calculated from the deconvolution of the areas using Equation (2) [26], as follows:

(2) $$Crl\;(\%)=\frac{A_{cryst}}{A_{total}}\times 100,$$

where *A_cryst_* is the sum of the crystalline areas, and *A_total_* is the total area under the diffractogram.

The lateral crystallite size (*L*_200_) was determined using Scherrer’s Equation (3) [27]:

(3) $$L_{200}=\frac{k\times \lambda }{\beta \times Cos\theta },$$

where *L*_200_ is the crystallite size (nm), *k* is the Scherrer constant (0.96), *λ* is the X-ray wavelength, *β* is half the full width at half maximum (FWHM) of the (200) reflection in radians, and θ is the Bragg angle corresponding to the (200) plane.

#### 2.3.6. Thermal Stability Analysis of Nanoparticles

Thermogravimetric analysis (TGA) of P-CNC was performed to evaluate the thermal stability and decomposition behavior of the samples. Thermogravimetric analysis was performed using a STA 6000 simultaneous thermal analyzer (PerkinElmer Inc., Waltham, MA, USA) at a heating rate of 10 °C min^−1^ from 25 to 600 °C under a nitrogen atmosphere (flow rate = 90 mL min^−1^). About 20 mg of each sample was analyzed. The Pyris software (version 13.4.0.0036) was used for data acquisition.

## 3. Results and Discussion

### 3.1. Influence of Reaction Conditions on Surface Charge and Zeta Potential on P-CNC Production

Table 1 shows the different combinations of reaction conditions and the measured responses, including the surface charge and zeta potential. The surface charge values obtained in this study ranged from 757 to 1993 mmol kg^−1^, which is within the expected range for phosphorylated cellulose nanocrystals synthesized in the presence of urea. Additionally, the zeta potential values were greater than −30 mV for all samples, with a maximum value of −67.40 mV. These results suggest that the P-CNC particles exhibit high colloidal stability in aqueous solution, which is consistent with previous studies [3,14], which reported comparable zeta potential values for phosphorylated CNCs.

Table 1 shows the surface charges of P-CNCs at different HPO_3_ concentrations and reaction times. The observed differences in surface charge can be directly attributed to the chemistry of phosphorylation. The hydroxyl groups on the main chain of cellulose, primarily at the C6 position, react with metaphosphoric acid to form negatively charged phosphate ester groups [28]. Longer reaction times and higher acid concentrations increase the density of these surface phosphate groups, resulting in more negative zeta potentials. Conversely, at high acid concentrations, the hydroxyl groups become saturated, and excessive phosphorylation can lead to the formation of polyphosphate or cross-links, limiting further increase in surface charge [9]. This chemical explanation clarifies the nonlinear trend observed in Table 1 and directly relates the reaction conditions to the measured surface charge of P-CNCs.

Initially, the surface charge increased and then decreased at high HPO_3_ concentrations, showing a nonlinear pattern. This trend highlights the interaction between reaction time and HPO_3_ concentration in the P-CNCs obtained. The results of the analysis of variance were used to evaluate the effects of reaction time and HPO_3_ concentration on the surface charge of the P-CNCs. The overall model was statistically significant (*p*-value = 0.0038, adjusted R^2^ = 0.92), indicating that at least one of the factors evaluated had a relevant effect on the response variables (Table S1, Supplementary Information). In particular, the reaction time had a highly significant effect (*p*-value = 0.0022), confirming its strong influence on the degree of substrate surface phosphorylation. HPO_3_ concentration also had a statistically significant effect (*p*-value = 0.0203), but to a lesser extent. In addition, a significant interaction was observed between both factors, time reaction × HPO_3_ concentration (*p*-value = 0.0097), suggesting that the effect of one factor depended on the level of the other factor. These results demonstrate that both the individual and combined reaction conditions significantly influenced the surface charge of the P-CNCs, with time being the most important factor.

Kröger et al. [14] demonstrated a methodology that allows the reliable and reproducible extraction of phosphorylated CNCs with exceptionally high surface charges (1920 mmol kg^−1^) using an improved reaction sequence. Another study demonstrated that highly charged phosphorylated CNCs with a surface charge of 1788 mmol kg^−1^ could be produced using a lower concentration of urea (4 M) and metaphosphoric acid [3]. Gao et al. [29] reported that a higher surface charge content, reaching up to 2330 mmol kg^−1^, improved the colloidal properties of CNCs. This finding aligns with the results of the present study, which showed that reaction time had a statistically significant effect on the surface charge. However, it is important to note that the surface charge does not depend exclusively on the reaction time; other parameters, such as temperature and reagent concentration, also play significant roles in the CNC phosphorylation.

The ANOVA conducted to evaluate the effects of reaction time and HPO_3_ concentration on the zeta potential (Table S2, Supplementary Information) indicated that only the reaction time had a statistically significant effect (*p*-value = 0.0488, adjusted R^2^ = 0.61). This result suggests that variations in the reaction time significantly influenced the zeta potential of the samples. In contrast, HPO_3_ concentration did not significantly affect the zeta potential, indicating that its influence on the zeta potential was negligible within the tested concentration range. Furthermore, the interaction between the two factors (time and HPO_3_ concentration) was not significant (*p*-value = 0.6978), suggesting that the effect of reaction time on the zeta potential was independent of the acid concentration. Although the overall model was not statistically significant, this was primarily due to the minimal contribution of HPO_3_ concentration and the interaction term, as the reaction time alone accounted for a substantial portion of the observed variability in the model.

Although the effect of HPO_3_ concentration on the surface charge of CNC was significant, its magnitude was relatively modest, and in the zeta potential analysis, the contribution of HPO_3_ concentration was not statistically significant, suggesting that this limited response can be explained by the near saturation of hydroxyl groups on CNCs, which reduces the scope for additional interactions at high acid concentrations. The phosphorylation of nanocellulose primarily proceeds through esterification at the C-6 hydroxyl group, which is attributed to its higher nucleophilic reactivity [28]. However, NMR analyses conducted by Lemke et al. [30] revealed that the hydroxyl groups at both the C-2 and C-6 positions can participate in the phosphorylation process. Once these sites are fully phosphorylated, additional acid does not change the surface charge. Phosphoric acid hydrolysis is known to improve the stability and thermal properties of CNCs; however, the charge and colloidal stability remain lower than those of CNCs hydrolyzed with sulfuric acid, indicating a limit to the effects of increased phosphoric acid concentration [9,21]. Additionally, while phosphoric acid can change the surface properties of CNCs’, zeta potential measurements can be influenced by several physicochemical parameters, including pH, ionic strength, and particle size. However, under controlled experimental conditions, these variables can be minimized to isolate the effects of other factors, such as reaction time. Understanding these dynamics is important for optimizing CNC production and achieving the desired material properties for various applications in the field.

**Figure 1:**
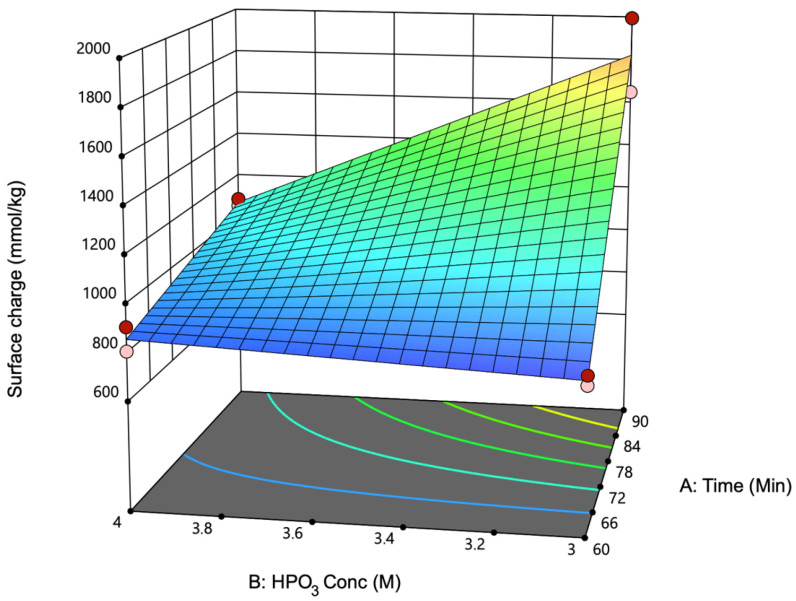
Response surface plot showing the combined effects of reaction time (A) and metaphosphoric acid concentration (B) on the surface charge of phosphorylated cellulose nanocrystals (P-CNCs). Color scale indicates the magnitude of the surface charge, with blue representing lower values and red representing higher values. The surface charge increased with both variables, reaching maximum values at the highest time (90 min) and acid concentration (4.0 M). The fitted model indicates a positive synergistic interaction between time and HPO_3_ concentration, suggesting that both parameters contribute significantly to phosphate group incorporation and surface functionalization.

Response surface methodology is commonly employed to represent the behaviors of static linear systems. It is characterized by a function that demonstrates how the output response is affected by various factors, enabling the prediction of outputs for factors that are not included in the initial design [31]. Figure 1 shows a three-dimensional response surface plot, demonstrating that increasing both the reaction time and HPO_3_ concentration led to a higher surface charge in the P-CNCs. This trend suggests that longer reaction times and higher acid concentrations allow extensive phosphorylation of the cellulose surface [3]. However, excessive phosphorylation can adversely affect the properties of CNCs. While moderate phosphorylation improves the thermal stability of CNCs compared to sulfated CNCs, excessive phosphorylation can significantly reduce the thermal stability of nanocrystals [9,32], likely due to the increased acid-catalyzed degradation of the cellulose chains at higher phosphate loadings. Furthermore, excessive phosphorylation can lead to the formation of polyphosphates and cross-linked phosphate groups on the CNC surface [14,33]. Such degradation can affect the mechanical and thermal properties of nanocrystals, potentially limiting their applicability in specific fields. Overall, the suitability of the factor models was assessed in each analysis performed; the R^2^ and adjusted R^2^ values are provided in the Supplementary Information (Tables S1 and S2), reinforcing the validity of the conclusions from this analysis.

### 3.2. Characterization of P-CNCs Obtained

#### 3.2.1. Morphological Analysis by TEM

**Figure 2:**
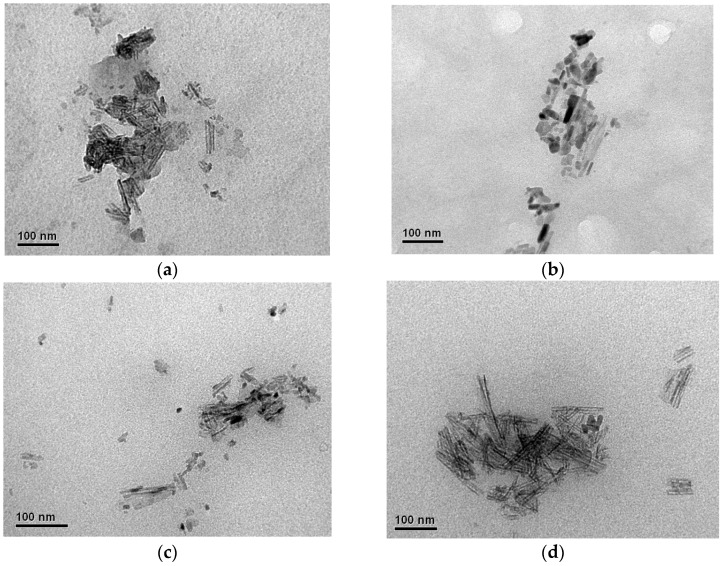
TEM images of the phosphorylated CNCs obtained in this study. The samples correspond to: (**a**) M1; (**b**) M2; (**c**) M3 and (**d**) M4. Scale bar: 100 nm.

The TEM images of the obtained samples revealed elongated rod-like nanostructures, which are consistent with the typical morphology of cellulose nanocrystals (Figure 2). This observation confirmed that the acid hydrolysis process successfully removed the amorphous regions of cellulose, leading to the formation of crystalline domains. The dimensions and shapes of the nanocrystals suggest that the reaction conditions and nature of the starting cellulose material play key roles in determining the final morphology of the nanocrystals.

**Figure 3:**
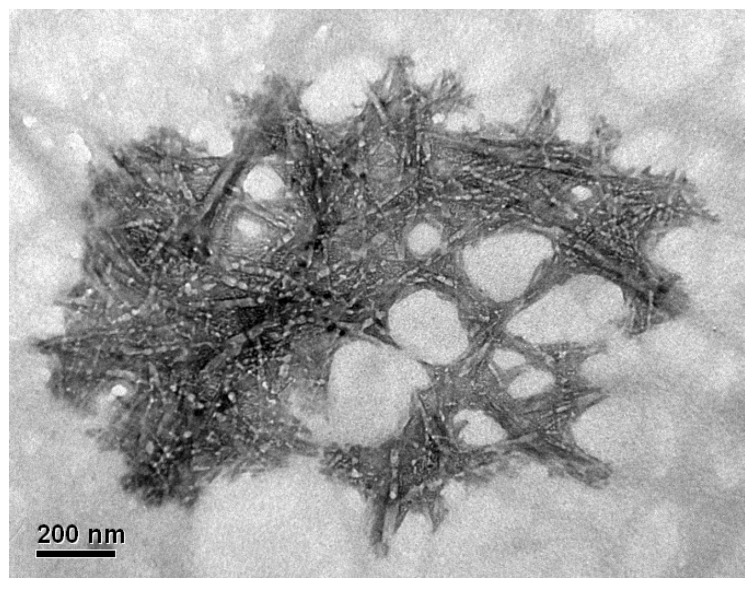
TEM images of the phosphorylated CNCs from sample M1, net-like structures from agglomerated P-CNCs. Scale bar: 200 nm.

The modification of the nanocrystal structure by incorporating new functional groups, such as phosphate groups, generates a negative charge that favors electrostatic repulsion between the particles, reducing their tendency to agglomerate [24]. In addition, agglomerations and dark patterns were clearly observed in the TEM images of the obtained CNCs, which were attributed to the high density of the phosphate groups or highly agglomerated regions and the concentration of particles in a suspension [34]. It has been reported in the literature that increasing the concentration of the phosphorylating agent in the CNCs isolation stage, using between 5 and 11 M H_3_PO_4_, produces greater agglomeration of CNC due to high densities of phosphate groups [35,36]. A similar behavior was observed for samples M4 (Figure 2d) and M2 (Figure 2b). Samples with higher acid concentrations exhibited pronounced dark patterns and thicker rods, which adopted a more cylindrical configuration and even formed networks owing to CNC agglomeration [36], as shown in Figure 3 for M1 sample.

#### 3.2.2. FTIR Functional Group Analysis of P-CNCs

**Figure 4:**
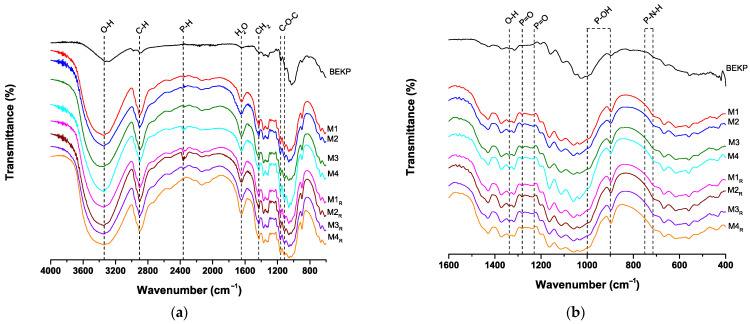
FTIR spectra of phosphorylated cellulose nanocrystals and unmodified bleached eucalyptus kraft pulp. (**a**) Full spectral range (4000–400 cm^−1^), showing typical cellulose vibrational bands. (**b**) Zoomed-in region (1600–400 cm^−1^), highlighting the emergence of new absorption bands related to phosphate group incorporation, such as P=O, P–OH, and P–N–H vibrational modes.

Figure 4 shows the FTIR spectra of the P-CNCs and BEKP. In Figure 4a, the spectra from 4000 to 400 cm^−1^ show the characteristic cellulose absorption bands. The broad band around 3340 cm^−1^ is attributed to the O–H stretching vibrations of hydroxyl groups, while the band at 2903 cm^−1^ corresponds to aliphatic C–H stretching [3,10,37]. A change in the O–H stretching vibration was observed in the P-CNC samples compared to the BEKP samples. In the case of P-CNC, the substitution of hydroxyl groups with phosphate groups modifies the pattern of hydrogen bonding interactions within the material. This has a direct impact on the O–H stretching vibration observed in spectroscopy studies, as the chemical environment around the hydroxyl groups is fundamentally altered [33]. Such changes can lead to shifts in the band position characteristic of O–H stretches when compared to unmodified cellulose materials like BEKP. The absorption at 1643 cm^−1^ was assigned to the bending vibrations of the absorbed water molecules. Notably, the band near 1430–1440 cm^−1^, related to CH_2_ symmetric bending and associated with cellulose I crystalline domains, exhibited increased intensity in the CNC samples, indicating an increase in crystallinity due to the removal of amorphous regions during acid hydrolysis [8,37]. The bands at 1160 and 1114 cm^−1^ were assigned to C–O–C stretching vibrations within the pyranose ring [3].

The band at 2364 cm^−1^, associated with P–H stretching vibrations, further confirmed the exposure of the cellulose substrate to phosphoric acid during phosphorylation [3,37,38]. Figure 4b provides a detailed view of the 1600–400 cm^−1^ region, where additional features resulting from the phosphorylation are evident. The band at 1338 cm^−1^ was assigned to the altered O–H deformation modes due to partial substitution with phosphate groups [37]. New bands appeared at 1282 and 1230 cm^−1^, which were attributed to P=O stretching vibrations [37,39,40]. Furthermore, the absorption bands observed between 900 and 1000 cm^−1^ are associated with P–OH stretching, which is indicative of phosphoric acid esterification [38]. The presence of a band in the 715–750 cm^−1^ range may be associated with the incorporation of phosphate groups into the cellulose matrix, as previously reported. Additionally, the inclusion of urea in the phosphorylation reaction is believed to promote the formation of P–N–H bonds, which is consistent with the appearance of vibrational bands in the same region [3,41].

#### 3.2.3. Hydrodynamic Behavior and Crystalline Structure of P-CNCs

Dynamic Light Scattering (DLS) is a widely employed technique for determining the hydrodynamic diameter of colloidal particles, such as cellulose nanocrystals (CNCs). This measurement encompasses not only the crystalline core of the particles but also the surrounding solvation layer and any transient agglomerates that may form in the suspension [42,43]. In this study, DLS analysis provided insights into the size distribution and colloidal behavior of the phosphorylated CNCs. Among the evaluated formulations, M3 and M4 exhibited the smallest average hydrodynamic diameters of 39.67 and 34.40 nm, respectively. In contrast, samples M1_R_ and M2_R_ had significantly larger particle sizes of 59.13 and 62.50 nm, respectively (Table 2).

The increase in hydrodynamic size from M1 to M2, corresponding to an increase in HPO_3_ concentration from 3 to 4 M with a reaction time of 60 min (Table 1), can be explained by the fact that, at low concentrations, CNC surfaces are only partially covered by phosphate groups; as the concentration increases, a higher density of phosphates is generated at the available sites, but the coverage remains insufficient to produce significant electrostatic repulsion, favoring the formation of weak aggregates and a larger size observed by DLS. Conversely, for longer reaction times (90 min, M3–M4), the density of phosphate groups on the surface is sufficient to increase electrostatic repulsion, overcoming the tendency to aggregate and resulting in smaller, well-dispersed particles; the prolonged time allows for a higher degree of conversion in the esterification process, while the temperature facilitates molecular mobility, preventing the formation of bridges between particles and reducing the hydrodynamic size [33,44]. The nonlinear behavior observed depends on the interaction between HPO_3_ concentration, reaction time, and temperature, where low concentrations and short times favor aggregation, while higher concentrations and longer times increase phosphate group coverage, generate electrostatic repulsion, and improve P-CNC dispersion.

The differences observed in hydrodynamic behavior indicate variations in colloidal stability, which may be influenced by surface charge, degree of phosphorylation, and ionic strength of the medium. Smaller particle sizes are often associated with higher colloidal stability, mainly due to increased electrostatic repulsion between the particles. This is usually reflected in higher (more negative) zeta potentials. Consistent with this trend, samples M3 and M4, which had the smallest particle sizes, also exhibited higher absolute zeta potentials (Table 1), suggesting better dispersion and a lower tendency to agglomerate. This inverse relationship between particle size and colloidal stability has been reported in previous studies. Vanderfleet et al. [9] found that phosphorylated CNCs with smaller hydrodynamic diameters (163 nm) exhibited more negative zeta potentials (−17.3 mV), whereas larger particles (246 nm) were less stable (−9.8 mV). Similarly, Kargarzadeh et al. [44] observed that longer acid hydrolysis times yielded smaller CNCs with reduced thermal stability but improved dispersion, whereas shorter hydrolysis durations led to larger, more thermally stable CNCs with lower colloidal stability (zeta potential near −10 mV).

**Figure 5:**
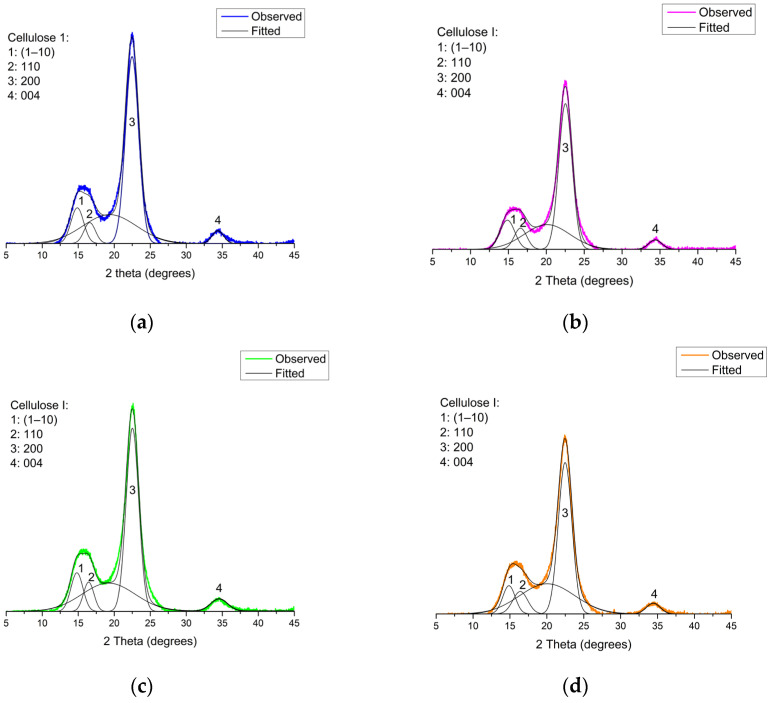
XRD P-CNC diffractograms obtained. (**a**) curve fitting of sample M1; (**b**) curve fitting of sample M2; (**c**) curve fitting of sample M3; and (**d**) curve fitting of sample M4.

The characterization of the supramolecular structure of the obtained phosphorylated cellulose nanocrystals and X-ray diffractograms (XRD) after peak deconvolution are presented in Figure 5 and its replicate in Figure S1 (Supplementary Information). All samples showed relatively high crystallinity indices, ranging from 63.6% to 71.3% (Table 2), indicating that the phosphorylation process partially preserved the crystalline domains of native cellulose I [45], and crystallite size (*L*_200_) between 4.21 nm and 4.64 nm. In more detail, the diffractograms showed characteristic peaks at 2θ values of approximately 14.8°, 16.5°, and 22.5°, which were assigned to the crystallographic planes (1–10), (110), and (200), respectively, which are typical of cellulose I polymorph. In addition, the reflection at 34.5° 2θ corresponds to the (004) plane of cellulose Iβ [46]. In contrast, sharper peaks can be observed at 22.5° (2θ), indicating that the difference in crystallinity between the samples is the most intense and sharpest peak (Figure 5a,c) with the highest degree of crystallinity, which can be attributed to the different acid concentrations and times employed [47]. Samples M2 and M4 (Figure 5b,d) showed peaks of lower intensity at 22.5°, likely due to the longer reaction times and higher reagent concentrations. Under these conditions, the acid can act as a solvent, promoting cellulose depolymerization and derivatization, which may reduce its crystallinity [48].

The crystallinity index values of the P-CNCs obtained in this study were lower than those reported in the literature [3,37]. Etale et al. [3] observed values ranging from 83% to 90% under similar phosphorylation conditions; this discrepancy can be primarily attributed to differences in the structural and chemical composition of the raw material. Specifically, the use of bleached hardwood kraft pulp, unlike highly purified sources, may result in the persistence of residual hemicelluloses and partially degraded cellulose chains even after extensive bleaching [49], contributing to a lower crystalline fraction in the final product [50]. Hemicelluloses and amorphous cellulose domains can mask or shield cellulose chains, preventing effective acid contact and subsequent hydrolysis, thereby reducing process efficiency [51]. This structural complexity can limit the removal of amorphous regions and reduce the formation of well-defined nanocrystals, thereby lowering the crystallinity index of the material. Additionally, the efficiency of surface functionalization, such as phosphorylation, can be affected by the accessibility of reactive hydroxyl groups, which are modulated by fiber purity and molecular organization [3].

These structural differences ultimately influence not only the crystallinity but also the morphological and colloidal behaviors of CNCs, which are critical parameters for their performance in advanced material applications. High-purity cellulose sources yield CNCs with high crystallinity and surface modification, which is desirable in fields such as biomedicine and electronics [52]. Bleached hardwood kraft pulp remains an attractive option for scalable, cost-effective applications such as flame-retardant coatings and barrier films, where moderate crystallinity and functionalization levels may be sufficient [50,53].

#### 3.2.4. Thermal Stability and Degradation Profile of P-CNCs

**Figure 6:**
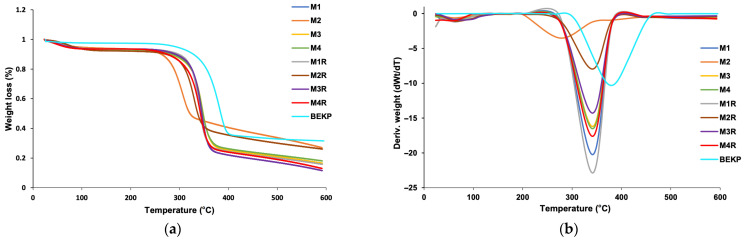
Thermogravimetric analysis of P-CNC and bleached hardwood kraft pulp (BEKP): (**a**) TG curves showing weight loss as a function of temperature, and (**b**) DTG curves representing the rate of weight loss.

Thermogravimetric analysis (TGA) and its derivative (DTG) revealed differences in the thermal behavior of P-CNC, samples M1–M4 and its replicates compared to BEKP (Figure 6). The observed differences in thermal degradation can be directly related to the degree of phosphorylation, particle morphology, and pathways of char formation, as discussed below. For P-CNC, one of the most notable characteristics was the early onset of mass loss observed at approximately 260 °C, which was attributed to dehydration reactions and the initial formation of carbonaceous charring. This process is favored by the presence of phosphate groups, which catalyze dehydration and hinder the rapid thermal decomposition of phosphate esters, thereby improving the overall thermal stability of the material [3,37].

The DTG curves (Figure 6b) show a single well-defined degradation peak for all samples, ranging from approximately 305 °C to 350 °C. Sample M2 had the lowest maximum degradation temperature (~305 °C), whereas samples M3 and M4 showed peaks closer to 347 °C (Table 3). The presence of a single major decomposition event supports the formation of cellulose phosphate as the dominant structure rather than mixtures such as ammonium phosphate and cellulose, which typically exhibit multiple degradation stages [3]. Samples M1, M3, and M4 showed relatively similar maximum degradation temperatures (~345 °C), whereas sample M2 decomposed at significantly lower temperatures, indicating structural or compositional differences likely related to a higher degree of phosphorylation or a different phosphate distribution.

Notably, phosphorylation resulted in a considerable increase in the residual mass at 600 °C, particularly in samples M2 and M2_R_, which retained 25.02% and 25.46% of their original mass, respectively. These values contrast with the lower residues observed in M1 and M1_R_ (9.54% and 14.48%, respectively), suggesting a lower degree of phosphate incorporation. Sample M3_R_ also showed greater carbon formation (22.03%), followed by M3 (15.21%), reinforcing the effectiveness of the phosphate groups in promoting thermal stability and carbon yield (Table 3). This behavior is consistent with the condensed-phase mechanism typically observed in phosphorylated polysaccharides, where phosphate groups act as dehydration catalysts, generating phosphoric and polyphosphoric acids that promote cyclization and aromatization reactions, resulting in thermally stable carbon structures [14,37,54].

Compared to sulfate-functionalized CNCs, which typically degrade at lower temperatures [9], the P-CNCs analyzed in this study exhibited a more gradual and prolonged degradation profile, indicating greater thermal resistance. Variations in carbon yield and degradation temperatures among P-CNCs may also be influenced by structural parameters such as particle morphology, size distribution, and accessibility of reactive hydroxyl groups [5,21]. Overall, the higher thermal stability and carbon yields observed in specific P-CNC samples, especially M2 and M3_R_, highlight their potential as flame-retardant additives in polymer composites, coatings, or other bio-based functional materials where improved fire resistance is desired.

## 4. Conclusions

This study successfully demonstrated the synthesis of phosphorylated cellulose nanocrystals (P-CNCs) from bleached hardwood kraft pulp (BEKP) under varying reaction conditions. The use of a metaphosphoric acid—urea system enabled efficient in situ phosphorylation, producing nanocrystals with tunable surface charges and excellent colloidal stability. Among the variables studied, the reaction time had the most significant effect on both the surface charge and zeta potential, where the acid concentration showed a moderate but relevant influence (surface charge ranging from 757 to 1993 mmol kg^−1^, zeta potential from −30.17 to −67.40 mV). The resulting P-CNCs exhibited rod-like morphologies characteristic of cellulose nanocrystals, with their dimensions determined by the reaction parameters. Fourier-transform infrared analysis confirmed the incorporation of phosphate groups, and X-ray diffraction (XRD) indicated the retention of the cellulose I crystalline structure. Thermal analysis revealed improved stability (Tmax 305–347 °C) and higher char yields (14.48–25.46% residual mass at 600 °C) compared to unmodified cellulose, supporting the potential of P-CNCs as flame-retardant additives in polymeric materials. These findings highlight the potential of BEKP as a sustainable raw material for the scalable production of functional nanocellulose materials. Although its lower purity compared to other sources may result in moderate crystallinity, the material properties obtained are suitable for applications in flame-retardant coatings, polymer composites, and barrier films.

By advancing the understanding of how reaction parameters influence the surface functionalization and thermal behavior of P-CNCs, this study provides valuable insights for the design of cellulose-based nanomaterials. Future research should explore the mechanical performance of composite matrices, the scalability of the production process, and the techno-economic feasibility of industrial applications.

## Figures and Tables

**Table 1:** Experimental matrix showing the reaction conditions and response variables: surface charge, particle size, and zeta potential of the P-CNCs.

Exp	Reaction Time (min)	HPO_3_ (M)	Surface Charge (mmol kg^−1^)	Zeta Potential (mV)
M1	60	3	757.29	−42.00 ± 0.96
M1_R_	60	3	797.92	−46.27 ± 1.56
M2	60	4	808.70	−30.17 ± 1.52
M2_R_	60	4	909.82	−55.63 ± 0.71
M3	90	3	1993.93	−59.67 ± 0.50
M3_R_	90	3	1653.47	−63.37 ± 0.90
M4	90	4	1057.03	−61.07 ± 0.50
M4_R_	90	4	1089.29	−67.40 ± 0.71

**Table 2:** Physicochemical characteristics of P-CNCs: hydrodynamic particle size, crystallinity index, and crystallite size (*L*_200_).

Exp	Particle Size (nm) *	Crystallinity Index (%)	Crystallite Size *L*_200_ (nm)
M1	45.17 ± 4.10	68.2	4.21
M1_R_	59.13 ± 2.64	71.3	4.41
M2	47.50 ± 1.15	66.6	4.42
M2_R_	62.50 ± 5.76	68.4	4.64
M3	39.67 ± 3.52	68.9	4.42
M3_R_	38.03 ± 4.29	67.8	4.64
M4	34.40 ± 3.22	63.6	4.21
M4_R_	35.87 ± 7.13	65.3	4.64

* Measurement by DLS.

**Table 3:** Maximum degradation temperature and residual mass for P-CNCs.

Exp	Maximum Degradation Temperature (°C)	Residue (%) at 600 °C
M1	345.4	19.54
M1_R_	305.9	25.02
M2	347.0	15.21
M2_R_	346.8	16.99
M3	341.7	14.48
M3_R_	329.9	25.46
M4	346.7	22.03
M4_R_	342.1	10.61

## Data Availability

The original contributions presented in this study are included in the article. Further inquiries can be directed to the corresponding author.

## Supplementary Materials

The following supporting information can be downloaded at: https://www.mdpi.com/article/10.3390/polym17192581/s1, Table S1: ANOVA to evaluate the effect of reaction time and metaphosphoric acidconcentration on charge density; Table S2: ANOVA to evaluate the effect of reaction time and metaphosphoric acid concentration on zeta potential; Figure S1: XRD P-CNC diffractograms obtained. (a) curve fitting of sample M1_R_; (b) curve fitting of sample M2_R_; (c) curve fitting of sample M3; and (d) curve fitting of sample M4.

## References

[1] Yang Y., Chen Z., Zhang R., Wang G., Suo D., Zhang J. (2019). Preparation and Applications of the Cellulose Nanocrystal. Int. J. Polym. Sci.

[2] Calle-Gil R., Castillo-Martínez E., Carretero-González J. (2022). Cellulose Nanocrystals in Sustainable Energy Systems. Adv. Sustain. Syst..

[3] Etale A., Onyianta A.J., Eloi J.C., Rowlandson J., Eichhorn S.J. (2024). Phosphorylated cellulose nanocrystals: Optimizing production by decoupling hydrolysis and surface modification. Carbohydr. Polym..

[4] Dagnino E.P., Ehman N., Area M.C. (2025). Recent Advances in Cellulose Nanocrystal Production from Green Methods. Processes.

[5] Lin N., Dufresne A. (2014). Surface chemistry, morphological analysis and properties of cellulose nanocrystals with gradiented sulfation degrees. Nanoscale.

[6] Li M.-C., Wu Y., Song K., Lee S., Qing Y., Wu Q. (2015). Cellulose Nanoparticles: Structure-Morphology-Rheology Relationships. ACS Sustain. Chem. Eng..

[7] Jordan J.H., Condon B.D., Easson M.W. (2019). Alkali Hydrolysis of Sulfated Cellulose Nanocrystals: Optimization of Reaction Conditions and Tailored Surface Charge. Nanomaterials.

[8] Aguayo M.G., Fernández-Pérez A., Reyes G., Oviedo C., Gacitúa W., Gonzalez R., Uyarte O. (2018). Isolation and Characterization of Cellulose Nanocrystals from Rejected Fibers Originated in the Kraft Pulping Process. Polymers.

[9] Vanderfleet O.M., Osorio D.A., Cranston E.D. (2018). Optimization of cellulose nanocrystal length and surface charge density through phosphoric acid hydrolysis. Phil. Trans. R. Soc. A..

[10] Aguayo M.G., Fernández-Pérez A., Oviedo C., Reyes G., Reyes-Contreras P. (2020). Relationship between Structural Characteristics of Cellulose Nanocrystals Obtained from Kraft Pulp. Nanomaterials.

[11] Soleimani S., Heydari A., Fattahi M. (2022). Isolation and Characterization of Cellulose Nanocrystals from Waste Cotton Fibers Using Sulfuric Acid Hydrolysis. Starch-Stärke.

[12] Huang S., Zhou D., Wu Q., Zhou L., Li M.-C. (2017). Cellulose Nanocrystals (CNCs) from Corn Stalk: Activation Energy Analysis. Materials.

[13] Maddalena L., Indias J.M., Bettotti P., Scarpa M., Carosio F. (2023). Cellulose nanocrystals polyelectrolyte complexes as flame retardant treatment for cotton fabrics. Polym. Degrad. Stab..

[14] Kröger M., Badara O., Kontturi E., Hietala S., Schlapp-Hackl I., Pääkkönen T. (2023). Efficient isolation method for highly charged phosphorylated cellulose nanocrystals. Biomacromolecules.

[15] Domingues R.M.A., Reis R.L., Gomes M.E. (2014). The potential of cellulose nanocrystals in tissue engineering strategies. Biomacromolecules.

[16] Sunasee R., Hemraz U. (2018). Synthetic Strategies for the Fabrication of Cationic Surface-Modified Cellulose Nanocrystals. Fibers.

[17] Arockiasamy F.S., Manoharan B., Santhi V.M., Prakalathan K., Periasamy D., Dhandapani A., Natarajan V., Krishnasamy S., Thiagamani S.M.K., Ilyas R.A. (2025). Navigating the nano-world future: Harnessing cellulose nanocrystals from green sources for sustainable innovation. Heliyon.

[18] Zhang Y.H.P., Cui J., Lynd L.R., Kuang L.R. (2006). A Transition from Cellulose Swelling to Cellulose Dissolution by *o*-Phosphoric Acid: Evidence from Enzymatic Hydrolysis and Supramolecular Structure. Biomacromolecules.

[19] Sathitsuksanoh N., Zhu Z., Zhang Y.H.P. (2012). Cellulose solvent-based pretreatment for corn stover and avicel: Concentrated phosphoric acid versus ionic liquid [BMIM] Cl. Cellulose.

[20] Ait Benhamou A., Sehaqui H., Nadifiyine M., Salim M.H., El Achaby M., Kassab Z., Moubarik A. (2021). Extraction, characterization and chemical functionalization of phosphorylated cellulose derivatives from Giant Reed Plant. Cellulose.

[21] Vanderfleet O.M., Reid M.S., Bras J., Heux L., Godoy-Vargas J., Panga M.K., Cranston E.D. (2019). Insight into thermal stability of cellulose nanocrystals from new hydrolysis methods with acid blends. Cellulose.

[22] Lu Q., Tang L., Wang S., Lin F., Cai Z., Huang B. (2016). Extraction of Cellulose Nanocrystals with a High Yield of 88% by Simultaneous Mechanochemical Activation and Phosphotungstic Acid Hydrolysis. ACS Sustain. Chem. Eng..

[23] Wang M., Yin G.Z., Yang Y., Fu W., Palencia J.L.D., Zhao J., Wang N., Jiang Y., Wang D.Y. (2023). Bio-based flame retardants to polymers: A review. Adv. Ind. Eng. Polym. Res..

[24] Camarero Espinosa S., Kuhnt T., Foster E.J., Weder C. (2013). Isolation of thermally stable cellulose nanocrystals by phosphoric acid hydrolysis. Biomacromolecules.

[25] Saito T., Isogai A. (2004). TEMPO-mediated oxidation of native cellulose. The effect of oxidation conditions on chemical and crystal structures of the water-insoluble fractions. Biomacromolecules.

[26] Park S., Baker J.O., Himmel M.E., Parilla P.A., Johnson D.K. (2010). Cellulose crystallinity index: Measurement techniques and their impact on interpreting cellulase performance. Biotechnol. Biofuels.

[27] Scherrer P. (1918). Bestimmung der Grosse und inneren Struktur von Kolloidteilchen mittels Röntgenstrahlen. Nachr. Ges. Wiss. Göttingen.

[28] Etale A., Onyianta A.J., Turner S.R., Eichhorn S.J. (2023). Cellulose: A review of water interactions, applications in composites, and water treatment. Chem. Rev..

[29] Gao X., Zhang L., Cui M., Huang R., Qi W., Su R. (2023). Pre-phosphorylation for facile production of phosphorylated cellulose nanocrystals with high charge content: An optimised design and life cycle assessment. Green Chem..

[30] Lemke C.H., Dong R.Y., Michal C.A., Hamad W.Y. (2012). New Insights into Nano-Crystalline Cellulose Structure and Morphology Based on Solid-State NMR. Cellulose.

[31] Varaksin A.N., Panov V.G., Katsnelson B.A., Minigalieva I.A. (2018). Using various nonlinear response surfaces for mathematical description of the type of combined toxicity. Dose-Response.

[32] Leszczyńska A., Szefer E., Mičušík M., Omastová M., Pielichowski K., Radzik P. (2019). Surface modification of cellulose nanocrystals with succinic anhydride. Polymers.

[33] Zhao M., Fujisawa S., Saito T. (2021). Distribution and quantification of diverse functional groups on phosphorylated nanocellulose surfaces. Biomacromolecules.

[34] Liu P., Borrell P.F., Božič M., Kokol V., Oksman K., Mathew A.P. (2015). Nanocelluloses and their Phosphorylated Derivatives for Selective Adsorption of Ag^+^, Cu^2+^ and Fe^3+^ from Industrial Effluents. J. Hazard. Mater..

[35] Phan-Xuan T., Bordes R., Labrador A., Matic A., Thuresson A., Skepö M. (2016). Aggregation behavior of aqueous cellulose nanocrystals: The effect of inorganic salts. Cellulose.

[36] Gan I., Chow W.S. (2021). Tailoring chemical, physical, and morphological properties of sugarcane bagasse cellulose nanocrystals via phosphorylation method. J. Nat. Fibers.

[37] Kokol V., Božič M., Vogrinčič R., Mathew A.P. (2015). Characterisation and properties of homo- and heterogenously phosphorylated nanocellulose. Carbohydr. Polym..

[38] Suflet D.M., Chitanu G.C., Popa V.I. (2006). Phosphorylation of polysaccharides: New results on synthesis and characterisation of phosphorylated cellulose. React. Funct. Polym..

[39] Bahloul A., Semlali F.Z., Oumam M., Hannache H., Kassab Z., El Achaby M. (2023). Starch bio-nanocomposites based on phosphorylated and sulphated cellulose nanocrystals extracted from pepper plant residue: Effect of surface functionality on property improvements. Cellulose.

[40] Belasri A., Blilid S., El Assimi T., Lahcini M., El Kadib A., Beniazza R. (2025). Soft phosphorylation of cellulose and starch for effective remediation of methylene blue dye and heavy metal-contaminated water. Int. J. Biol. Macromol..

[41] Petreuş O., Bubulac T., Petreuş I., Cazacu G. (2003). Reactions of some phosphorus compounds with cellulose dissolved in aqueous alkaline solution. J. Appl. Polym. Sci..

[42] Pecora R. (2000). Dynamic light scattering measurement of nanometer particles in liquids. J. Nanopart. Res..

[43] Zhang C., Jin Z., Zeng B., Wang W., Palui G., Mattoussi H. (2020). Characterizing the Brownian diffusion of nanocolloids and molecular solutions: Diffusion-ordered NMR spectroscopy vs dynamic light scattering. J. Phys. Chem. B.

[44] Kargarzadeh H., Ahmad I., Abdullah I., Dufresne A., Zainudin S.Y., Sheltami R.M. (2012). Effects of hydrolysis conditions on the morphology, crystallinity, and thermal stability of cellulose nanocrystals extracted from kenaf bast fibers. Cellulose.

[45] Carrillo-Varela I., Retamal R., Pereira M., Mendonça R.T. (2019). Structure and reactivity of cellulose from bleached kraft pulps of different Eucalyptus species upgraded to dissolving pulp. Cellulose.

[46] Kusmono, Affan M.N. (2022). Isolation and characterization of nanocrystalline cellulose from ramie fibers via phosphoric acid hydrolysis. J. Nat. Fibers.

[47] Alemdar A., Sain M. (2008). Isolation and characterization of nanofibers from agricultural residues–wheat straw and soy hulls. Bioresour. Technol..

[48] Zhang J., Zhang J., Lin L., Chen T., Zhang J., Liu S., Li Z., Ouyang P. (2009). Dissolution of Microcrystalline Cellulose in Phosphoric Acid—Molecular Changes and Kinetics. Molecules.

[49] Hernandez C.C., Ferreira F.F., Rosa D.S. (2018). X-ray powder diffraction and other analyses of cellulose nanocrystals obtained from corn straw by chemical treatments. Carbohydr. Polym..

[50] Rampazzo R., Alkan D., Gazzotti S., Ortenzi M.A., Piva G., Piergiovanni L. (2017). Cellulose nanocrystals from lignocellulosic raw materials, for oxygen barrier coatings on food packaging films. Packag. Technol. Sci..

[51] Rajan K., Djioleu A., Kandhola G., Labbé N., Sakon J., Carrier D.J., Kim J.W. (2020). Investigating the effects of hemicellulose pre-extraction on the production and characterization of loblolly pine nanocellulose. Cellulose.

[52] Börjesson M., Westman G. (2015). Crystalline Nanocellulose—Preparation, Modification, and Properties. Cellulose: Fundamental Aspects and Current Trends.

[53] Ramires E.C., Dufresne A. (2011). A review of cellulose nanocrystals and nanocomposites. Tappi J..

[54] Aoki D., Nishio Y. (2010). Phosphorylated cellulose propionate derivatives as thermoplastic flame resistant/retardant materials: Influence of regioselective phosphorylation on their thermal degradation behaviour. Cellulose.

