# Supplementary material for: Synthesis and Characterization of Phosphorylated Cellulose Nanocrystals: Exploring Factors for Enhanced Thermal and Colloidal Stability
Source: Polymers (Basel). 2025 Sep 24;17(19):2581. doi: 10.3390/polym17192581 (PMC12526524; doi:10.3390/polym17192581)
Supplement: Supplementary file 1 [file polymers-17-02581-s001.zip › polymers-3853711-supplementary.pdf]

Synthesis and Characterization of Phosphorylated Cellulose Nanocrystals: Exploring Factors for Enhanced Thermal and Colloidal Stability

Diego López [1], María Graciela Aguayo P [1,2,*], Mario Núñez Decap [2,3], Pablo Reyes-Contreras [4], Regis Teixeira Mendonça [4,5], Isidora Reyes [5], Benjamín Opazo [1], and Fabiola Valdebenito [6,7]

1 Departamento de Ingeniería de Procesos y Bioproductos, Facultad de Ingeniería, Universidad del Bío-Bío, Avenida Collao 1202, Concepción 4051381, Chile; diego.lopez2001@alumnos.ubiobio.cl (D.L.); benjamin.opazo2001@alumnos.ubiobio.cl (B.O.)
2 Centro Nacional de Excelencia Para la Industria de la Madera (CENAMAD) - ANID BASAL FB210015, Pontificia Universidad Católica de Chile, Vicuña Mackenna 4860, Santiago 7820436, Chile; mnunez@ubiobio.cl (M.N.-D.)
3 Departamento de Ingeniería Civil y Ambiental, Facultad de Ingeniería, Universidad del Bío-Bío, Avenida Collao 1202, Concepción 4051381, Chile
4 Laboratorio de Biopolímeros y Materiales Biobasados, Centro de Biotecnología, Universidad de Concepción, Concepción 4030000, Chile; preyesc@udec.cl (P.R.-C.); rteixeira@udec.cl (R.T.M.)
5 Facultad de Ciencias Forestales, Universidad de Concepción, Concepción 4030000, Chile; ireyes2017@udec.cl (I.R.-G.)
6 Centro de Energía, Universidad Católica de la Santísima Concepción, Concepción 4090541, Chile; fvaldebenito@ucsc.cl (F.V.)
7 Departamento de Química Ambiental, Facultad de Ciencias, Universidad Católica de la Santísima Concepción, Concepción 4090541, Chile
* Correspondence: maguayo@ubiobio.cl (M.G.A.)

SUPPLEMENTARY INFORMATION

Table S1. ANOVA to evaluate the effect of reaction time and metaphosphoric acid concentration on charge density.

Table S2. ANOVA to evaluate the effect of reaction time and metaphosphoric acid concentration on zeta potential.

Figure S1. XRD P-CNC diffractograms obtained. (a) curve fitting of sample $M1_R$; (b) curve fitting of sample $M2_R$; (c) curve fitting of sample M3; and (d) curve fitting of sample M4.

Table S1. ANOVA to evaluate the effect of reaction time and metaphosphoric acid concentration on charge density.

Source	Sum of Squares	df	Mean Square	F-value	p-value
Model	1.364E+06	3	4.545E+05	28.24	0.0038
A: Time	7.937E+05	1	7.937E+05	49.32	0.0022
B: HPO_3 Conc.	2.237E+05	1	2.237E+05	13.90	0.0203
AB	3.462E+05	1	3.462E+05	21.51	0.0097
Pure Error	64377.44	4	16094.36	—	—
Total	1.428E+06	7	—	—	—
R^2 : 0.9549			Predicted R^2 : 0.8196		
Adjusted R^2 : 0.9211			Adeq Precision : 11.6580		

Table S2. ANOVA to evaluate the effect of reaction time and metaphosphoric acid concentration on zeta potential.

Source	Sum of Squares	df	Mean Square	F-value	p-value
Model	704.83	3	234.94	2.67	0.1830
A: Time	689.50	1	689.50	7.84	0.0488
B: HPO_3 Conc.	0.0055	1	0.0055	0.0001	0.9941
AB	15.32	1	15.32	0.1743	0.6978
Pure Error	351.59	4	87.90	—	—
Total	1056.42	7	—	—	—
R^2 : 0.6672			Predicted R^2 : 0.5313		
Adjusted R^2 : 0.6176			Adeq Precision : 5.2182		

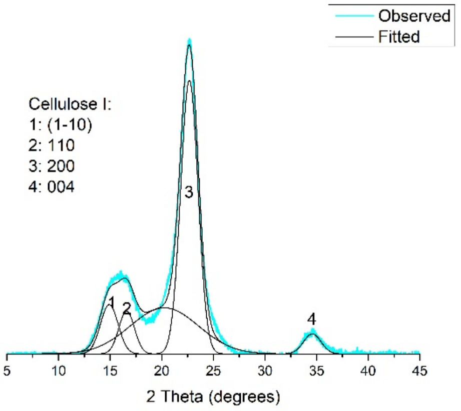

(**a**)

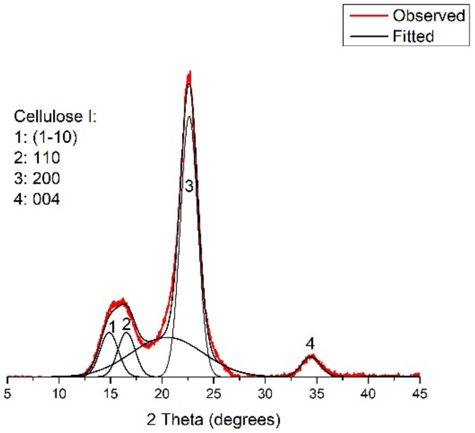

(**b**)

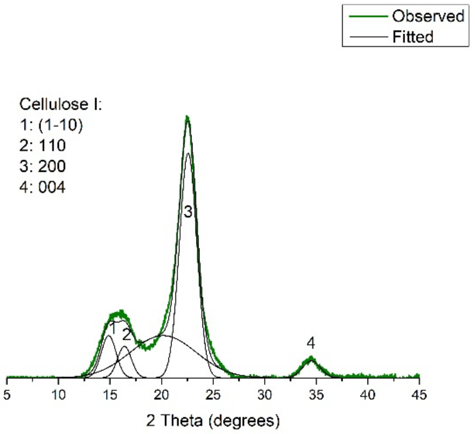

(**c**)

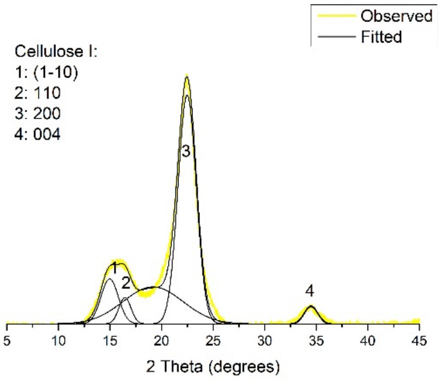

(**d**)

Figure S1. XRD P-CNC diffractograms obtained. (a) curve fitting of sample $M1_R$; (b) curve fitting of sample $M2_R$; (c) curve fitting of sample M3; and (d) curve fitting of sample M4.
